# Agomelatine improves memory and learning impairments in a rat model of LPS-induced neurotoxicity by modulating the ERK/SorLA/BDNF/TrkB pathway

**DOI:** 10.1007/s00210-023-02717-w

**Published:** 2023-09-15

**Authors:** Mahmoud Abdelaziz, Ahmed F. Mohamed, Hala F. Zaki, Sameh S. Gad

**Affiliations:** 1 Department of Pharmacology and Toxicology, Faculty of Pharmacy, October University for Modern Sciences and Arts (MSA University), Giza, Egypt; 2 Department of Pharmacology and Toxicology, Faculty of Pharmacy, Cairo University, Kasr El-Aini St, Cairo, 11562 Egypt; 3 Faculty of Pharmacy, King Salman International University (KSIU), 46612 Ras Sedr, South Sinai Egypt

**Keywords:** Agomelatine, ERK ½, SorLA, BDNF, Neuroplasticity, Autophagy

## Abstract

The mutual interplay between neuroinflammation, synaptic plasticity, and autophagy has piqued researchers’ interest, particularly when it comes to linking their impact and relationship to cognitive deficits. Being able to reduce inflammation and apoptosis, melatonin has shown to have positive neuroprotective effects; that is why we thought to check the possible role of agomelatine (AGO) as a promising candidate that could have a positive impact on cognitive deficits. In the current study, AGO (40 mg/kg/day, p.o., 7 days) successfully ameliorated the cognitive and learning disabilities caused by lipopolysaccharide (LPS) in rats (250 μg/kg/day, i.p., 7 days). This positive impact was supported by improved histopathological findings and improved spatial memory as assessed using Morris water maze. AGO showed a strong ability to control BACE1 activity and to rein in the hippocampal amyloid beta (Aβ) deposition. Also, it improved neuronal survival, neuroplasticity, and neurogenesis by boosting BDNF levels and promoting its advantageous effects and by reinforcing the pTrkB expression. In addition, it upregulated the pre- and postsynaptic neuroplasticity biomarkers resembled in synapsin I, synaptophysin, and PSD-95. Furthermore, AGO showed a modulatory action on Sortilin-related receptor with A-type repeats (SorLA) pathway and adjusted autophagy. It is noteworthy that all of these actions were abolished by administering PD98059 a MEK/ERK pathway inhibitor (0.3 mg/kg/day, i.p., 7 days). In conclusion, AGO administration significantly improves memory and learning disabilities associated with LPS administration by modulating the ERK/SorLA/BDNF/TrkB signaling pathway parallel to its capacity to adjust the autophagic process.

**Supplementary Information:**

The online version contains supplementary material available at 10.1007/s00210-023-02717-w.

## Introduction

Cognitive deficit refers to the loss of several intellectual abilities including attention, thinking, learning, and reasoning (Hsing et al. 2015). The progression of cognitive decline and memory deficits in a variety of neurological diseases has been linked to neuroinflammation. Neuroinflammation is a multifaceted process and a hallmark of almost every central nervous system disorder (Joshi et al. 2014).

Lipopolysaccharide (LPS) is considered an important tool in studying several neurological disorders related to cognitive functions, because of its propensity to activate toll-like receptors (TLRs) triggering a drastic immune response with a plethora of cellular and molecular immune components leading to neuroinflammation and consequentially deterioration of learning and memory (Joshi et al. 2014; Batista et al. 2019; Zhu et al. 2019). LPS administration additionally demonstrated modulated autophagy and impaired neuroplasticity which could also affect learning and memory (François et al. 2014; Zhu et al. 2019). That is why LPS is frequently utilized to evaluate the correlation between neuroinflammation, autophagy, neuroplasticity, and cognitive deficits in experimental sets. Previous studies showed that repeated LPS injections have the potential to elevate Aβ_1–42_ levels either by elevating amyloid precursor protein (APP) level or by enhancing the β-site amyloid precursor protein cleaving enzyme (BACE1) activity, thereby impeding cognitive capabilities (Lee et al. 2008a; Mohamed et al. 2021).

Protein 3D folding and clearance of misfolded proteins must be precise in order to maintain health because protein misfolding, aggregation, and accumulation may be a reason behind a range of several neurological disorders, e.g., Aβ accumulation (Bharadwaj et al. 2009; Samant and Frydman 2019). Comprehensive evidence has proved that misfolded protein accumulation due to deregulation of cellular mechanisms responsible for protein sorting and aggregates metabolism can cause synaptic dysfunction, neuronal death, brain damage, and as a consequence cognitive dysfunction and memory impairment (Soto and Estrada 2008; Herczenik and Gebbink 2008; Lu et al. 2017). Therefore, protein sorting to its exact location in the subcellular compartments is of high importance for optimal cellular function (Schmidt et al. 2017). A crucial regulator of this process is the Sortilin-related receptor with A-type repeats (SorLA), which directs cargo proteins, like kinases, and phosphatases to the proper location within the cell (Schmidt et al. 2017).

As an adaptive response to many harsh conditions, autophagy mediates a highly regulated self-eating process via lysosomes. It also plays a significant part in maintaining cellular equilibrium, either by removing damaged cells or by preventing the accumulation of proteins that are aggregated and have been folded incorrectly (He and Klionsky 2009). Therefore, excessive or inadequate levels could be harmful and cause damage (Chen et al. 2014).

Synaptic plasticity refers to the process by which the efficacy of a synapse is strengthened or weakened over time. As a result, synaptic plasticity is considered critical for proper cognitive abilities (Bourgognon and Cavanagh 2020; Goto 2022). Cognitive impairment is strongly linked to the loss of the presynaptic markers synaptophysin and synapsin I, as well as the postsynaptic marker PSD-95 (Sultana et al. 2010; Hajjar et al. 2013; Mirza and Zahid 2018).

Changes in neurotrophic factors levels, e.g., brain-derived neurotrophic factor (BDNF) is also linked to cognitive deficit (Budni et al. 2015). This always happens based on their ability to regulate several points related to cognition and the healthy brain including neurogenesis, synaptic plasticity, protein sorting, and autophagy (Tartaglia et al. 2001; Chen et al. 2017a; Nikoletopoulou et al. 2017). Loss of neurotrophic factors especially BDNF can lead to synaptic dysfunction, impaired autophagy, disturbed protein folding, and finally cognitive impairment (Chen et al. 2017a).

Extracellular signal-regulated kinase ½ (ERK ½) is considered of high importance when it comes to learning and memory, this could be related to its ability to support the formation of new dendritic spines, and the regulation of several synaptic proteins, maintaining long-term potentiation (LTP) (Peng et al. 2010; Albert-Gascó et al. 2020). In addition, it has a role in the transmission of cell signals from the cell surface to the nucleus, and this is important for cellular proliferation and differentiation (Peng et al. 2010). This pathway could be blocked by administering PD98059 a MEK/ERK inhibitor which prevents the activation of MEK by RAF, leading to sequential block of the ERK pathway (Wang et al. 2017).

Agomelatine (AGO) is a novel antidepressant and a structural analogue of melatonin with a distinct mode of action. Its therapeutic effects are not only mediated by binding to the melatonergic MT1 and MT2 receptors, but also by acting as a 5-HT2C receptor antagonist (Can et al. 2018). It was previously mentioned that melatonin can have a beneficial action in boosting memory and cognitive abilities (Mansouri et al. 2022). In this study, we attempted to shed light on the promising strategy that AGO could have against LPS-mediated cognitive decline by modulating many factors, e.g., neuroinflammation, amyloid beta deposition, autophagy, and synaptic plasticity mediated by the ERK ½ pathways, which could be confronted using PD98059.

## Materials and methods

### Experimental animals

In our study, adult male Wistar rats weighing 150 to 200 g. Animals were bought from the animal facility of the National Research Centre (Dokki, Cairo, Egypt). Then, they were housed in climate-controlled rooms with (25 ± 2 °C) temperature, (12:12 light–dark cycles), and controlled humidity (60 ± 5%). During the entire holding period, they had free access to water and commercial diet pellets. Prior to the experiment, animals were housed for a couple of weeks to accommodate; then, they were randomly distributed across the groups.

### Drugs and chemicals

We used LPS, *E. coli* [O111:B4] (Cat. No. L2630) purified by phenol extraction, PD98059 (Cat. No. P215) both from Sigma-Aldrich (St Louis, MO, USA), Also, we used agomelatine (Valdoxan, Servier laboratories). Other chemicals in this study were highly pure and solutions for chemicals and drugs were freshly prepared prior to use.

### Experimental design

A total of 32 male Wistar albino rats 150 to 200 gm were used. Animals were allocated at random into four groups (*n* = 8) after they were left for 1 week for acclimatization. The groups were treated as follows:

- Group 1 (control group): serves as a normal control group receiving saline for 7 days.
- Group 2 (LPS-only group): rats were treated with LPS (250 μg/kg) in saline intraperitoneally (I.P.) daily for 1 week (Khan et al. 2019; Adetuyi and Farombi 2021).
- Group 3 (LPS + AGO group): rats received LPS (250 μg/kg) in saline I.P. + AGO (40 mg/kg) in saline by oral gavage 3 h after each LPS injection for 7 days (Aslankoc et al. 2020).
- Group 4 (LPS + AGO + PD 98059 group): after 1 h of receiving I.P. dose of LPS (250 μg/kg), then PD98059 was administered at a dose of (0.3 mg/kg) I.P. in 10 mg/mL of 10% DMSO diluted in phosphate-buffered saline (Jin et al. 2017; Bian et al. 2020). Two hours later, a dose of AGO (40 mg/kg) in saline was given orally. The previously mentioned sequence was given for a successive 7 days.

### Behavioral analysis

#### Morris water maze (MWM)

Using MWM, we evaluated spatial memory retention, as well as the capacity of experimental animals to acquire and recall particular tasks (Adetuyi and Farombi 2021). The experiment was carried out in a standard pool (diameter = 1.5 m, depth = 50 cm), that was further divided into 4 main sections marked with a fixed visual cue at each pole North (N), South (S), East (E), and West (W) respectively. Water was stained in black using a non-toxic black food additive, and its temperature was settled at 25 ± 2 °C. In the center of the (W) section, a movable hidden platform was placed during the acquisition test which lasted 5 days starting from day 4 to day 7. The probe test was performed on the 8th day before the animals were euthanized. In the acquisition phase, animals were trained to search for the hidden platform. Rats were subjected to three daily trials from different starting points; each trial lasted up to 2 min. Rats that made it to the platform within time were left for 10 s before being removed; however, those who had not found it after 2 min were led there manually by hand to the hidden platform and left for half a minute. The time interval between each trial either in the acquisition phase and the probe phase vary from 3 to 30 min. The escape latency was then calculated by averaging the total time taken in all trials on each day. The platform was taken away on the 8th day, and each animal underwent a test by placings them in section (E), which is the opposite and farthest point from the standard position of the platform. For 1 min, each rat was allowed to swim freely across the pool, and we tracked how much time was spent in the target section by each animal (Sayed et al. 2020; Mohamed et al. 2021). To rule out the impact of visual, motor, and motivational variability on rat performance, a cued trial was conducted after the probe test. In this test, the escape platform was kept submerged, but we added a “flag” extending above the water surface by approximately 12 cm to direct the rats toward the platform.

### Hippocampal processing

On the 8th day, rats were euthanized under light anesthesia. Then, we harvested the brains and divided them into 2 subgroups. The brains of the 1st subgroup (*n* = 2) were isolated and fixed in 10% formalin before being histo-pathologically examined. Brains of the 2nd subgroup (*n* = 6), were dissected directly for hippocampal isolation and storage at – 80 °C. Following that, the left isolated hippocampi were subjected to a Western blot, and the right part was subjected to ELISA.

### Biochemical measurements

#### Enzyme-linked immunosorbent assay (ELISA)

The hippocampal homogenate was processed in phosphate-buffered saline [PBS] at a PH = 7.4 by the help of a polytron homogenizer at 4 °C, in order to assess some parameters. For the purpose of eliminating cell debris, the homogenate was centrifuged at 10,000 rpm for 20 min. The estimation of Aβ1-42 was performed using the supernatant. Using a protein estimation kit from Genei, Bangalore, the protein content of the tissue was ascertained using the method of Bradford (1976). ELISA plate reader was used to measure color absorbance from 490 to 630 nm (Stat Fax 2200, Awareness Technologies, Florida, USA). Fluoroskan Ascent Fluorometer microplate reader was used to measure all fluorescent kits (Thermo Fisher Scientific Oy FI-01621 Vantaa, Finland). Rat Aβ1-42 ELISA kit from LSBio (Seattle, WA, USA) was used to assess Aβ1-42 (Catalogue No: LS-F23254). Rat Beclin 1 (BECN1) ELISA kit from Biomatik (Delaware, USA) (Catalogue No: EKU02685), Rat MAP1LC3B/LC3B ELISA kit (Sandwich ELISA) from LifeSpan Biosciences, Inc. (Seattle, WA, USA) (Catalogue No: LS-F19802), and p62 ELISA kit from creative diagnostics (Ramsey Road Shirley, NY, 11,967, USA) (Catalogue No: DEIA6457) were used to assess Beclin 1, LC3B, and p62, respectively. Rat Sortilin-related receptor (SORL1) ELISA kit was used to assess SORL1 from MyBioSources (San Diego, California, USA) (Catalogue No: MBS7213656). Rat Syn1(Synapsin-1) ELISA kit (Wuhan, Hubei, China) (Catalogue No: ER0583), rat synaptophysin (SYP) ELISA kit from Abbexa Ltd. (Cambridge Science Park, Cambridge, UK) (Catalogue No: abx256618), and rat DLG4/PSD-95 ELISA kit from LifeSpan BioSciences, Inc. (Seattle, WA, USA) (Catalogue No: LS-F6865) were used respectively to assess synapsin I, synaptophysin, and PSD-95. Beta-secretase activity was determined using BACE-1 activity assay kit (Fluorometric) obtained from Novus Biologicals (Catalogue No: KA0900). The Fluoroskan Ascent Fluorometer microplate reader was used to obtain the results (Thermo Fisher Scientific Oy FI-01621 Vantaa, Finland). All according to the manufacturer’s recommendations. Beta-amyloid 1–42 (Aβ1-42) and synapsin I were expressed in picogram per milligram protein, while SORL1, synaptophysin, PSD-95, LC3B, Beclin 1, p62 were expressed in nanogram per milligram protein. BACE-1 activity was expressed in microgram per milligram protein.

#### Western blot

Following the manufacturer’s instructions, each homogenized sample of all different groups was added to the ReadyPrep protein extraction kit (total protein) from Bio-Rad Inc (Catalogue No. 163–2086). The Bradford protein assay kit (SK3041) for quantitative protein analysis from Bio Basic Inc. (Markham Ontario L3R 8T4 Canada). The protein concentration of each sample was measured using the Bradford assay based on the manufacturer’s recommendations. Then, an equal volume of 2 × Laemmli sample buffer containing 4% SDS, 10% 2-mercaptoethanol, 20% glycerol, 0.004% bromophenol blue, and 0.125 M Tris HCl was loaded onto each sample at a 20 μg protein concentration at PH of 6.8. Before loading on polyacrylamide gel electrophoresis, each previous mixture was boiled at 95 °C for 5 min to ensure denaturation of the protein. The TGX Stain-Free™ FastCast™ Acrylamide Kit (SDS-PAGE) from Bio-Rad Laboratories Inc. under catalogue number: 161–0181 was used to create polyacrylamide gels. The manufacturer’s instructions were followed on preparing the SDS-PAGE TGX Stain-Free FastCast. From below to above, the gel was assembled in a transfer sandwich (filter paper, PVDF membrane, gel, and filter paper). The sandwich was then transferred and added to a transfer tank containing 1 × transfer buffer (25 mM Tris, 190 mM glycine, and 20% methanol). The blot was then run at 25 V for 7 min to allow protein bands to be transferred from gel to membrane using the Bio-Rad Trans-Blot Turbo. At room temperature, the membrane was blocked in tris-buffered saline with Tween 20 (TBST) buffer and 3% bovine serum albumin (BSA) for 1 h. The blocking buffer contained the following ingredients: 20 mM Tris pH 7.5, 150 mM sodium chloride, 0.1% Tween 20, and 3% BSA, and primary antibodies of anti-BDNF (EPR1292) antibody (Catalogue No: ab108319, 1:1000 dilution; Abcam), anti-Phospho-ERK1/2 (Thr202/Tyr204) antibody (Catalogue No: A1589-100, 1:1000 dilution; Biovision), anti-ERK1 + ERK2 (EPR17526) antibody (catalogue No: ab184699, 1:10,000 dilution; Abcam), and anti-TrkB (EPR17805-146) antibody (Catalogue No: ab187041, 1:5000 dilution; Abcam). According to manufacturer’s recommendations, TBST was used to dilute the primary antibodies.

Each primary antibody solution was incubated against the blotted target protein for an entire night at 4 °C. The blot was rinsed with TBST 3–5 times for 5 min. The blotted target protein was incubated in the HRP-conjugated secondary antibody (Goat anti-rabbit IgG-HRP-1 mg Goat mab-Novus Biologicals) solution for a full hour at room temperature then the blot was rinsed with TBST. As instructed by the manufacturer, the chemiluminescent substrate (ClarityTM Western ECL substrate Bio-Rad cat#170–5060) was applied to the blot after rinsing with TBST 3–5 times for 5 min. Membranes were stripped for reprobing with new antibody by incubating them in stripping buffer. Briefly, equal volumes from Clarity Western luminal/enhancer solution (solution A) and peroxidase solution (solution B) were used. The chemiluminescent signals were monitored and recorded using a CCD camera-based imager. The band intensity of the targeted proteins was read against beta-actin in the control sample utilizing software for image analysis after protein normalization on the ChemiDoc MP imager.

### Histopathological findings

Brain samples were preserved for 24 h in 10% buffered formol saline, then dehydrated in alcohol, cleared in xylene, and then integrated in paraffin wax. Under a light microscope (Olympus BX50, Tokyo, Japan), sections of 4–5 μm stained with hematoxylin and eosin (H&E) were examined in order to reveal any histopathological alteration.

### Statistical analysis

The statistical analysis was done using one-way analysis of variance test (ANOVA) followed by Tukey’s test for multiple comparisons, with exception of the time spent during the training test of MWM, which was analyzed using two-way analysis of variance followed by Tukey’s test using GraphPad Prism (Version 9) at *p* < 0.05.

## Results

### Effect of AGO on hippocampal* Aβ *deposition,* BACE1 *activity, and SorLA levels

**Fig. 1:**
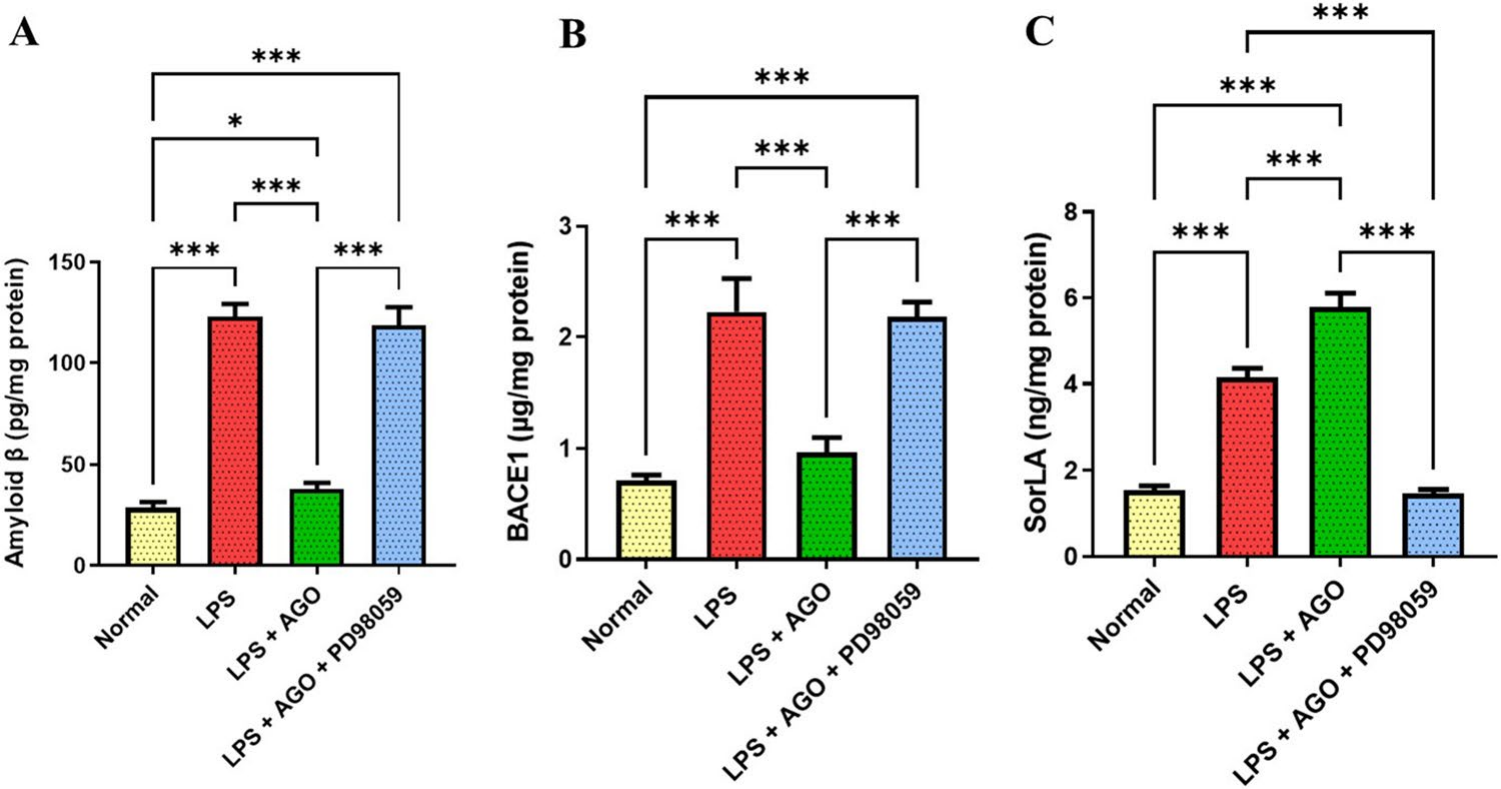
Effect of AGO on hippocampal *Aβ* deposition, *BACE1* activity, and SorLA levels. Lipopolysaccharide was administrated daily for 1 week (250 μg/kg, I.P.) in order to induce deterioration of memory and cognitive abilities each group except for the control. Meanwhile, AGO (40 mg/kg, p.o.) was administrated daily 3 h after each LPS dose for 1 week in group 3 and group 4, finally PD98059 (0.3 mg/kg, I.P.) in DMSO and PBS in the 4th group 1 h after each LPS injection followed by AGO after 2 h. PD98059 administration abolished the effect of AGO in suppressing Aβ deposition in LPS-induced cognitive deficit in rats. **A** Also, PD98059 attenuated the beneficial effect of AGO in minimizing BACE1 activity. **B** SorLA levels was also assessed using ELISA showing a significant elevation in its expression in presence of AGO in comparison to LPS and this action was diminished in presence of the blocker. **C** All values are presented as the mean ± S.D. and statistical analysis was carried out using one-way analysis of variance test (ANOVA) followed by Tukey’s test for multiple comparisons. The *p*-value for significance was set at *p* < 0.05. *p < 0.05, ** < 0.01, ***p < 0.001

Following LPS administration, there was a significant increase in hippocampal activity of BACE1 and as a consequence in the Aβ levels by 2.13- and 3.31*-*folds compared to the control. On the contrary, AGO administration showed a significant reduction in their levels by 56.7% and 69.1% respectively in comparison to the group that received LPS only, and there was no significance difference between the BACE1 activity between the normal control group and the LPS + AGO group. Meanwhile, the beneficial action of AGO was suppressed in the group that was pretreated with PD98059 and this was shown by an elevation in BACE1 activity and Aβ levels by 1.27- and 2.13-folds respectively in comparison to the group that only received AGO. Concerning SorLA levels, the LPS-only group showed a significant increase in SorLA level by 1.69 compared to the control group. In addition, when AGO and LPS were administered concurrently, the SorLA level significantly increased by 39.2% in comparison to the LPS-only group. In addition, pre-administering PD98059 showed a significant reduction in SorLA level by 74.9% (Fig. 1).

### Effect of AGO on hippocampal autophagy biomarkers

**Fig. 2:**
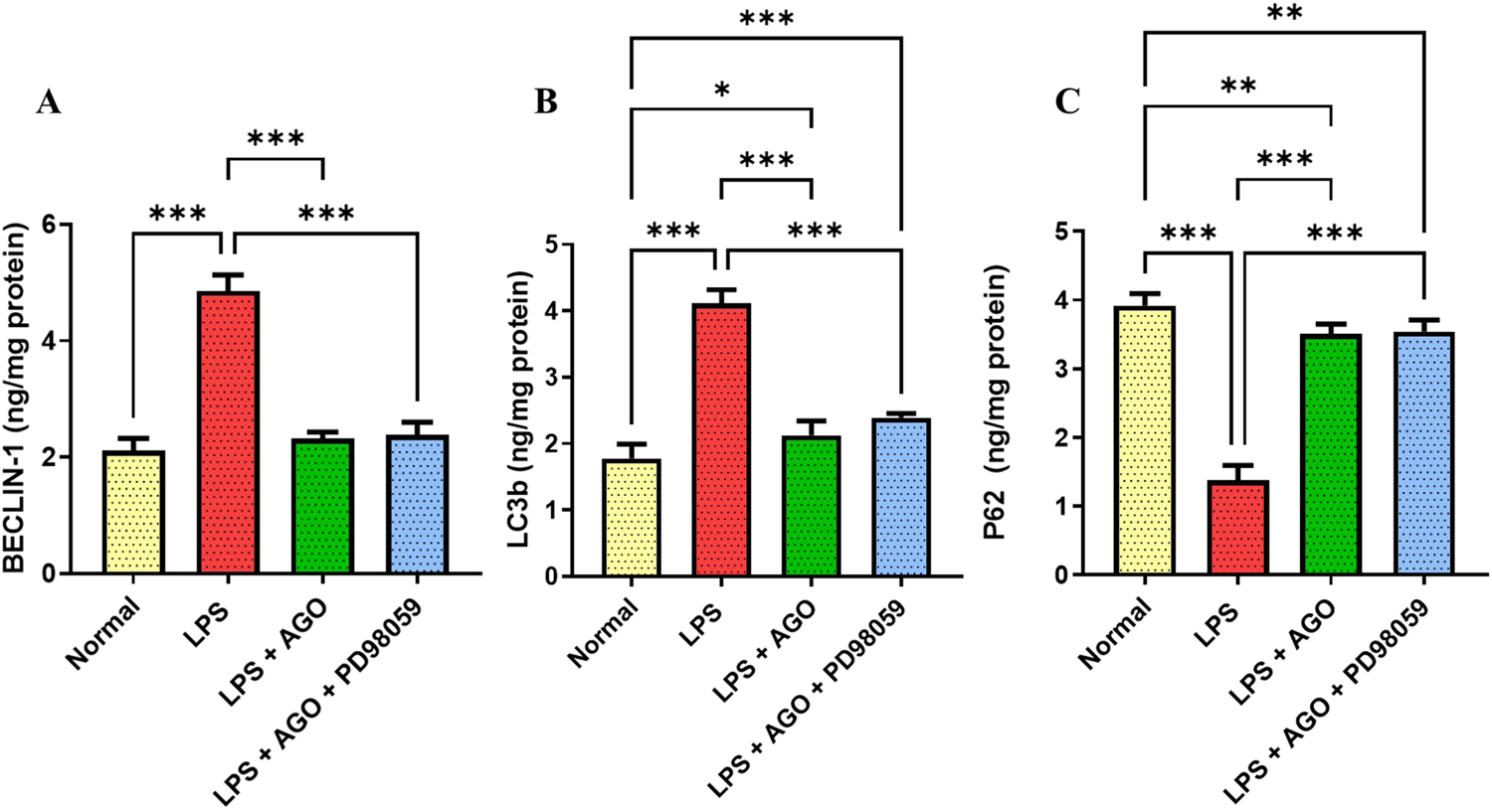
Effect of AGO on hippocampal autophagy biomarkers. Lipopolysaccharide was administrated daily for one week (250 μg/kg, I.P.) in order to induce deterioration of memory and cognitive abilities each group except for the control. Meanwhile, AGO (40 mg/kg, p.o.) was administrated daily 3 h after each LPS dose for 1 week in group 3 and group 4, finally PD98059 (0.3 mg/kg, I.P.) in DMSO and PBS in the 4th group 1 h after each LPS injection followed by AGO after 2 h. Administration of LPS only showed a significant elevation in Beclin 1 levels in comparison to the normal control group, on the other hand, AGO significantly reduced Beclin 1 level to normal in comparison to the LPS-only group. There was no significance difference between the LPS + AGO group in comparison to the LPS + AGO + PD98059 group. **A** LC3b levels was significantly increased in the LPS-only group in comparison to the control group, but its level was significantly reduced in presence of AGO in comparison to the LPS-only group. Administering PD98059 had no impact on the LC3b level in comparison to the LPS + AGO group. **B** Concerning the effect of AGO on P62 levels, it was significantly elevated in comparison to the LPS-only group, which showed a significant reduction in its level in the LPS-only group in comparison to the normal control group. Administering PD98059 showed no significant difference in comparison to the LPS + AGO group. **C** All values are presented as the mean ± S.D. and statistical analysis was carried out using one-way analysis of variance test (ANOVA) followed by Tukey’s test for multiple comparisons. The *p*-value for significance was set at *p* < 0.05. *p < 0.05, ** < 0.01, ***p < 0.001

Active autophagy was prevalent in the LPS-only group compared to the control group. This was demonstrated by elevated LC3b and Beclin1 levels by 1.32- and 1.29-folds respectively with a significant decrease in P62 level by 64.9%. On the contrary, AGO significantly reduced Beclin1 and LC3b levels by 52.1% and 48.3% respectively and elevated p62 levels by 1.55 times compared to the LPS-only group indicating inhibitory action on autophagy biomarkers. Also, the impact of adding PD98059 to the treatment group on the autophagy biomarkers was non-significant (Fig. 2).

### Effect of AGO on hippocampal *mBDNF*, *pTrkB* content, and *pERK/tERK* ratio

**Fig. 3:**
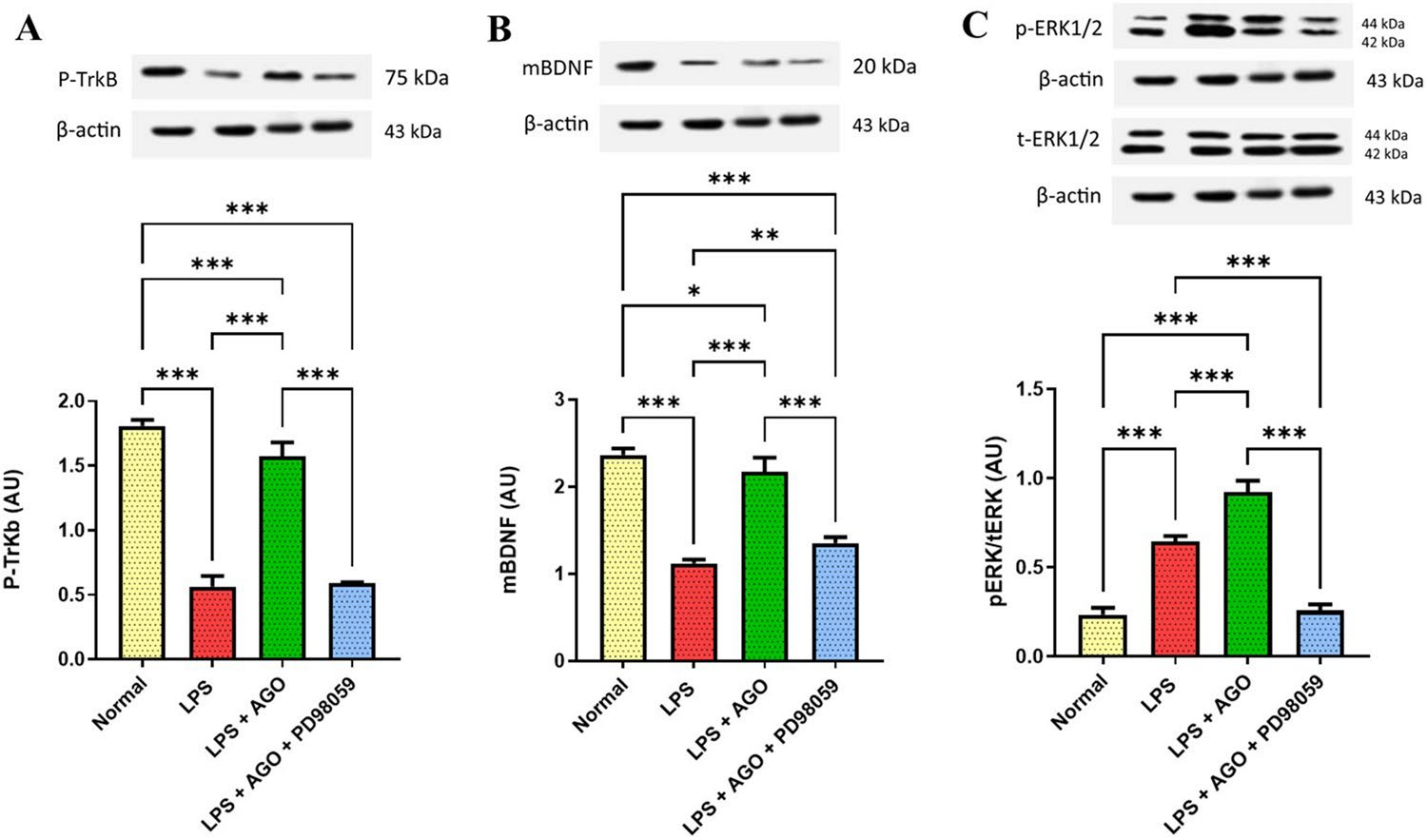
Effect of AGO on hippocampal *mBDNF*, *pTrkB* content, and *pERK*/*tERK* ratio*.* Lipopolysaccharide was administrated daily for one week (250 μg/kg, I.P.) in order to induce deterioration of memory and cognitive abilities each group except for the control. Meanwhile, AGO (40 mg/kg, p.o.) was administrated daily 3 h after each LPS dose for 1 week in group 3 and group 4, finally PD98059 (0.3 mg/kg, I.P.) in DMSO and PBS in the 4th group 1 h after each LPS injection followed by AGO after 2 h. Blockade of ERK using PD98059 negatively affected the beneficial effect of AGO in attenuating the deleterious effect of LPS on the expression of PTrkB, and mBDNF respectively in the hippocampal region. Administration of LPS-only showed a significant decrease in their expression in comparison to the control group. On the other hand, AGO significantly increased their expression in comparison to the LPS-only group, but this beneficial effect was significantly diminished in presence of PD98059 in comparison to the LPS + AGO group. This was also represented by using western blot (**A**, **B**). The effect of blocking ERK pathway using PD98059 in the hippocampal region was also assessed. LPS-only showed a significant increase the pERK/tERK ratio in comparison to the control group, also administering AGO significantly increased this ratio in comparison to either the control group or the LPS-only group. On the other hand, administering the blocker showed a significant decrease in comparison to the LPS-only group and the LPS + AGO groups (**C**). All values are presented as the mean ± S.D. and statistical analysis was carried out using one-way analysis of variance test (ANOVA) followed by Tukey’s test for multiple comparisons. The *p*-value for significance was set at *p* < 0.05. *p < 0.05, ** < 0.01, ***p < 0.001

Administering LPS showed a significant reduction in the pTrkB and mBDNF content by 52.7% and 68.8%, respectively, compared to control. Concomitant administration of AGO with LPS increased the BDNF content and pTrkB expression by 93.9% and 1.79-folds, respectively. Also, the group that received LPS + AGO + PD98059 showed a significant reduction in the previously mentioned parameters by 37.6% and 62.7% respectively. On the other hand, LPS-only group showed a significant increase in Perk/tERK ratio by 1.75-folds, compared to the control group. In addition, LPS + Ago administration showed a significant increase in the perk/tERK ratio by 43.1% respectively in comparison to the LPS-only group. In addition, pre-administering PD98059 showed a significant reduction in pERK/tERK ratio by and 72.1% (Fig. 3).

### Effect of AGO on hippocampal *Synaptophysin*, *PSD95*, and *Synapsin I* content

**Fig. 4:**
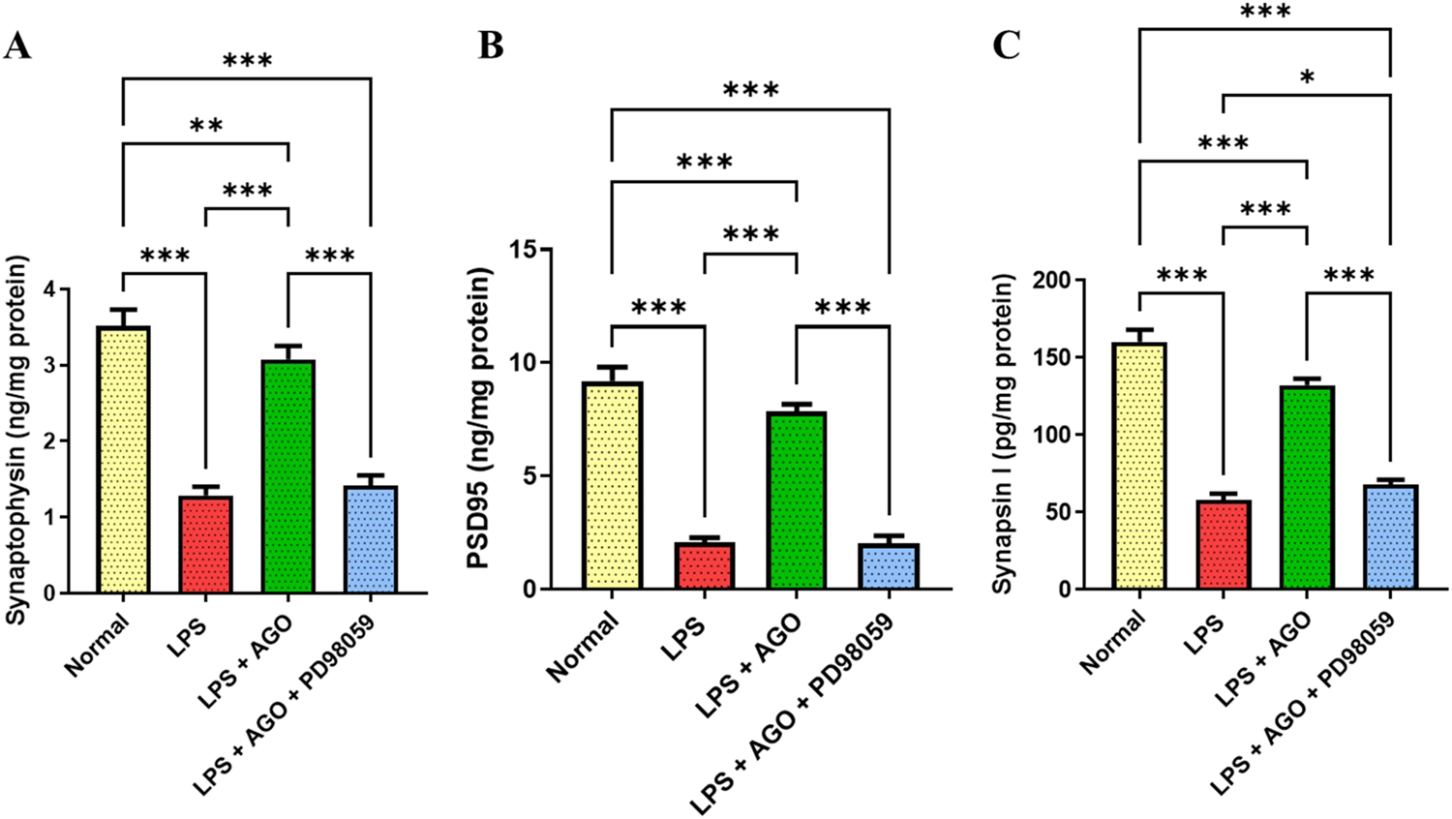
Effect of AGO on hippocampal *Synaptophysin*, *PSD95*, and *Synapsin I* content. Lipopolysaccharide was administrated daily for one week (250 μg/kg, I.P.) in order to induce deterioration of memory and cognitive abilities each group except for the control. Meanwhile, AGO (40 mg/kg, p.o.) was administrated daily 3 h after each LPS dose for 1 week in group 3 and group 4, finally PD98059 (0.3 mg/kg, I.P.) in DMSO and PBS in the 4th group 1 h after each LPS injection followed by AGO after 2 h. AGO significantly attenuated synaptic dysfunction, and significantly elevated the levels of the synaptic plasticity biomarkers (Synaptophysin, PSD-95, and Synapsin I) in comparison to the LPS-only group and this action was abolished in presence of PD98059 respectively. **A**, **B**, and **C** All values are presented as the mean ± S.D. and statistical analysis was carried out using one-way analysis of variance test (ANOVA) followed by Tukey’s test for multiple comparisons. The *p*-value for significance was set at *p* < 0.05. *p < 0.05, ** < 0.01, ***p < 0.001

LPS treatment showed a recognized decline in Synaptophysin, PSD95, and Synapsin I levels by 63.3%, 77.4%, and 63.7%, respectively compared to the control. Conversely, the LPS + AGO showed a significant and substantial increase in Synaptophysin, PSD95, and Synapsin I levels by 1.39-, 2.79-, and 1.28-folds, respectively compared to the LPS-only group. Additionally, compared to the LPS + AGO only, the previously mentioned parameters showed a significant decrease by 53.9%, 74.2%, and 48.5%, respectively when PD98059 was used (Fig. 4).

### Morris water maze

**Fig. 5:**
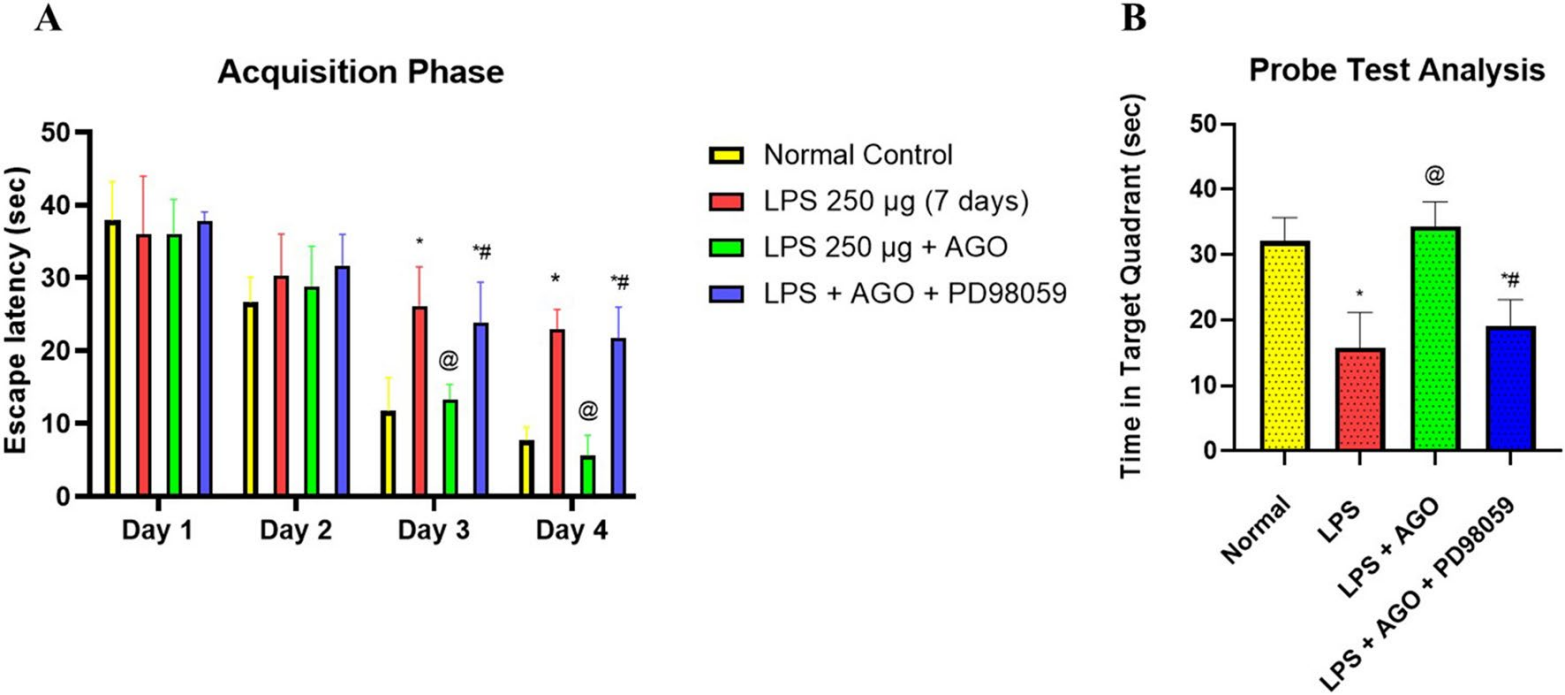
Lipopolysaccharide was administrated daily for one week (250 μg/kg, I.P.) in order to induce deterioration of memory and cognitive abilities each group except for the control. Meanwhile, AGO (40 mg/kg, p.o.) was administrated daily 3 h after each LPS dose for 1 week in group 3 and group 4, finally PD98059 (0.3 mg/kg, I.P.) in DMSO and PBS in the 4th group 1 h after each LPS injection followed by AGO after 2 h. Using MWM, we evaluated spatial memory retention. The experiment was carried out in a standard pool that was further divided into 4 main sections marked with a fixed visual cue at each pole. Water was stained in black using a non-toxic black food additive, and its temperature was settled at 25 ± 2 °C. A movable hidden platform was placed during the acquisition phase starting from day 4 to day 7. The probe phase was performed on the 8th day before the animals were euthanized. Results of behavioral analysis using MWM. The time of average escape latency for rats in order to find the platform was assessed starting from day 4 to day 7. There was no significant difference across the groups in the first 2 days of training. Starting from the 3rd and the 4th day, the group that received the LPS only showed a significant deterioration in the learning capabilities in comparison to the control group. On the other hand, the group that received LPS + AGO showed enhanced learning capabilities and the time needed to reach the platform was decreased in comparison to the LPS-only group. Administering PD98059 deteriorated the beneficial effect of AGO on the cognitive functions by increasing the time needed to find the hidden platform in comparison to the LPS + AGO group (**A**). Assessment of time spent in platform quadrant in the probe phase also took place on day 8, where the group that received LPS only showed a significant decrease in the time spent in the target quadrant in comparison to the normal control group, while the group that received LPS + AGO showed a significant increase in the time spent in the target quadrant compared to the LPS-only group. This time was significantly decreased on the concomitant use of PD98059 compared to the group that received LPS + AGO (**B**). All values are presented as the mean ± S.D. and statistical analysis was carried out using two-way analysis of variance to analyze the time spent during the training test of MWM, and one-way analysis of variance for the probe phase. Both were followed by Tukey’s test for multiple comparisons. The *p*-value for significance was set at < 0.05. *Significantly different from normal group. ^@^Significantly different from the LPS-only group. ^#^Significantly different from the LPS + AGO group

Concerning the MWM test, rats that received LPS showed learning disabilities along the training period starting from the 4th day of the experiment, but AGO significantly minimized this deteriorating action, where animals reached the platform successfully in a shorter time. This action was diminished in presence of PD98059. During the probe phase, the LPS-only group showed a significantly impaired spatial memory where the time spent in the specified section was decreased by 50.9% if compared to control group, thus showing impaired memory function. The time spent in the target section after AGO administration was significantly increased by 1.18-folds if compared to the group that received LPS only. This beneficial effect was reduced by 44.3% in comparison to the group that was treated with PD98059 (Fig. 5). In addition, to rule out the impact of visual, motor, and motivational variation on the tested groups performance, a cued trial was conducted after the probe test. In this test, there was no statistically significant difference in escape latencies between any of the groups.

### Histopathology

**Fig. 6:**
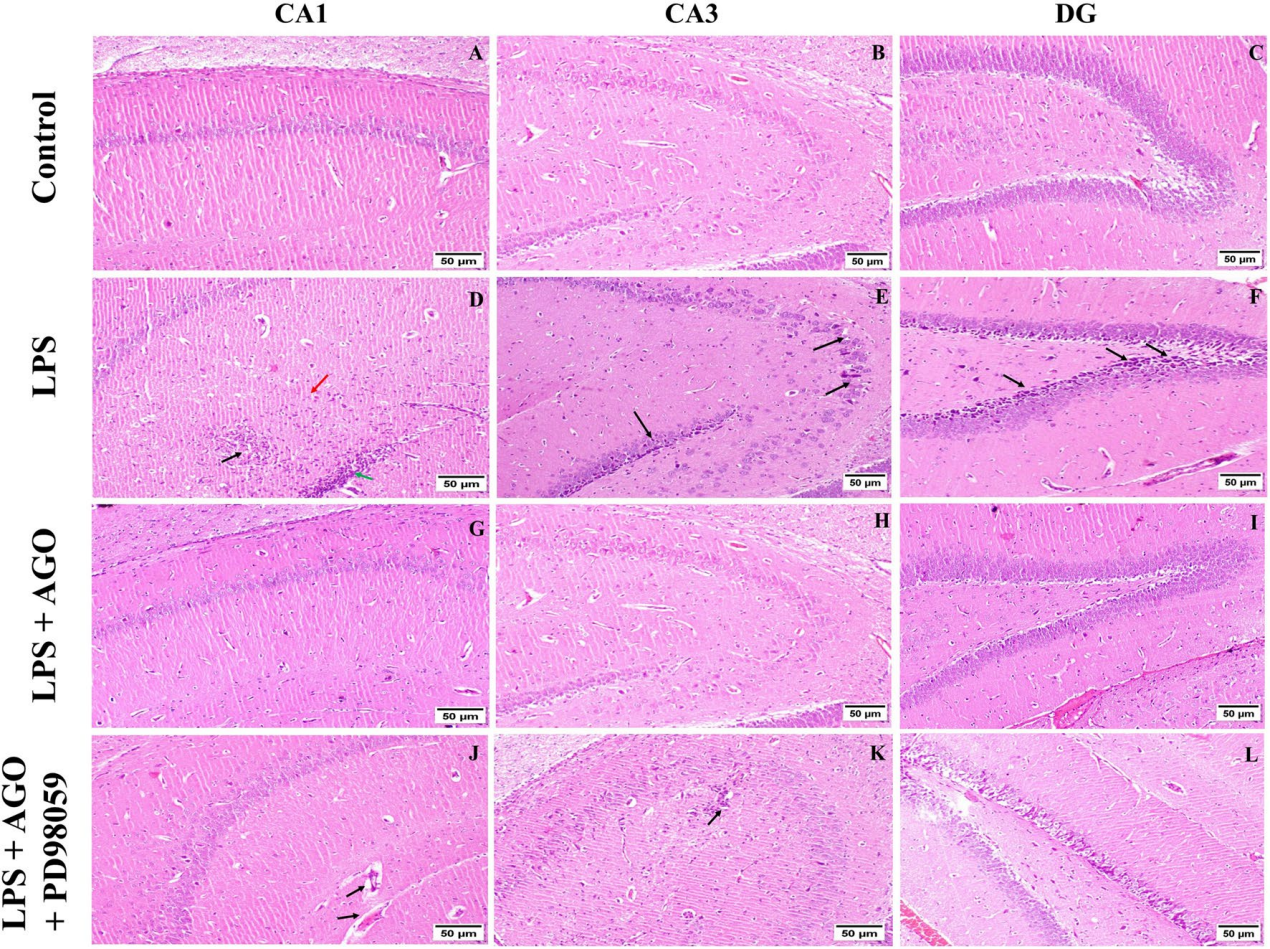
Indicative photomicrographs showing H&E staining of various hippocampus regions showed histologically normal hippocampal regions in the 1st group that received saline only (**A**, **B**, **C**). Concerning the LPS-only group, CA1 region showed area of malacia (black arrow), gliosis (red arrow) and lymphocytic infiltration (green arrow) as seen in (**D**). Also, dark degenerating neurons was prominent within the CA3 & DG regions (black arrows) as seen in (**E**, **F**). An apparently normal hippocampus was observed in the LPS + AGO group (**G**, **H**, **I**). Photomicrograph of the hippocampus of the group that received LPS + AGO + PD98059 showed mild thickening in the blood vessels wall of the CA1 region (black arrows) (**J**). Also, focal gliosis was seen in the CA3 region (black arrow), and mild neuronal degeneration and edema was prominent at the DG region as seen in (**K**, **L**) consecutively

Indicative photomicrographs showing H&E staining of various hippocampus regions showed histologically normal hippocampal regions in the 1st group that received saline only (Fig. 6A, B, C). Concerning the LPS-only group, CA1 region showed area of malacia (black arrow), gliosis (red arrow), and lymphocytic infiltration (green arrow) as seen in (Fig. 6D). Also, dark degenerating neurons were prominent within the CA3 & DG regions (black arrows) as seen in (Fig. 6E, F). An apparently normal hippocampus was observed in the LPS + AGO group (Fig. 6G, H, I). Photomicrograph of the hippocampus of the group that received LPS + AGO + PD98059 showed mild thickening in the blood vessels wall of the CA1 region (black arrows) (Fig. 6J). Also, focal gliosis was seen in the CA3 region (black arrow), and mild neuronal degeneration and edema were prominent at the DG region as seen in (Fig. 6K, L) consecutively.

## Discussion

Our results demonstrated that AGO successfully reversed LPS-induced neurocognitive deficits in rats. This impact is possibly attributed to AGO modulatory impact on ERK/SorLA/BDNF/TrkB pathway and its consequent influence on amyloidogenesis, synaptogenesis, neuroplasticity, and autophagy.

In the present study, systemic administration of LPS showed a detrimental effect on learning and memory. This action could result from neuroinflammation, amyloidogenesis, loss of neurotrophic factors, impaired neuroplasticity, and autophagy (Anaeigoudari et al. 2016; Frühauf-Perez et al. 2018; Abou El-ezz et al. 2018). Deposition of Aβ even at low concentrations might impair learning and memory as amyloidogenesis can disrupt several signaling pathways crucial for synaptic plasticity and neurogenesis (Chen et al. 2009; Ma and Klann 2012). In accordance with previous studies, administration of LPS showed an elevated BACE1 activity and increased Aβ level in the rats’ hippocampi, which is considered a precursor to the cognitive impairment and spatial memory dysfunction seen in this recent study (Lee et al. 2008b; Adetuyi and Farombi 2021). The histopathological picture further confirmed the damage that the LPS had on the hippocampus, as it led to prominent neuronal edema and degeneration within some hippocampal areas like dentate gyrus (DG), CA1, and CA3.

It was reported that MT1 agonists could have a positive impact in reducing amyloidogenesis, and BACE1 activity as well as the cognitive impairment induced by LPS (Panmanee et al. 2015; Sun et al. 2020). The current study found that AGO had a beneficial effect on reducing BACE1 activity and Aβ deposition, but this action was abolished in presence of PD98059. MWM was used in order to assess the potential behavioral changes that might take place among the four experimental groups. According to the findings, group 2 and group 4 spent less time in the targeted section during the probe phase and took longer time to find the escape platform during acquisition phase than group 1 or group 3.

We may infer from the above results that LPS administration led to learning and memory deficits. Additionally, we deduced that PD98059 impaired learning and spatial memory, negating the positive impacts of AGO treatment on memory function.

Numerous biological processes, such as cellular differentiation, proliferation, and death, rely on MAPK signaling pathway (Wang et al. 2017). One of the MAPK signal transduction pathways, the ERK 1/2 pathway is essential for LTP, synaptic remodeling, in addition to memory consolidation (Peng et al. 2010; Lim et al. 2014). Previous studies have shown that PD98059 administration inhibited hippocampal LTP, adversely impacted neuroplasticity, and impaired memory by inhibiting ERK1/2 phosphorylation (Blum et al. 1999; Medina and Viola 2018). In the current study, LPS boosted pERK/tERK ratio and SorLA expression in the hippocampal regions. Elevation of hippocampal pERK/tERK ratio in presence of LPS was previously documented and could be dose and time dependent (Zhao et al. 2017; Tong et al. 2019). LPS possibly increased SorLA expression via ERK activation, which could be a cytoprotective compensatory mechanism to protect against the drastic actions involved in the neurological changes that appeared after LPS administration, e.g., Aβ deposition (Gao et al. 2022). Administering AGO also strikingly elevated the phosphorylation of ERK and as a consequence increased SorLA expression*.* These results were also supported by previous studies where melatonin showed a positive impact on the ERK ½ phosphorylation rate (Xiong et al. 2015). Surprisingly, PD98059 was able to reduce SorLA expression. As a result, we hypothesized that AGO activates SorLA via the ERK signaling pathway.

In particular, SorLA can control amyloidogenesis. This relies on its capacity to reduce Aβ synthesis either by steering APP away from the early endosome toward the Golgi, preventing it from being processed to the neurotoxic Aβ in the late endocytic compartments or by directing newly formed Aβ molecules toward lysosomal degradation (Andersen et al. 2005; Rohe et al. 2009; Caglayan et al. 2014). Additionally, it was proposed that SorLA functions as a trafficking receptor to stop BACE1 cleavage of APP by preventing the interaction between BACE1 and APP (Spoelgen et al. 2006; Rohe et al. 2009). In this study, SorLA expression was upregulated along with marked decline in BACE1 activity and Aβ deposition, which could be attributed to the AGO action on SorLA expression, and this action was diminished by administering PD98059.

BDNF is one of the most abundant neurotrophins that has a great impact on neurogenesis, neuronal survival, neuroplasticity, learning, and memory (Pradhan et al. 2019; Yang et al. 2020b). These actions are related to the ability of the mature BDNF (mBDNF) to bind strongly to TrkB, activating some pathways, e.g., phosphatidylinositol 3-kinase (PI3K), MAPK/ERK, and PLCγ (Ahmed et al. 2021). It is important to note that BDNF production depends on several aspects, where BDNF can self-enhance its own level by activating the ERK pathway. It has previously been demonstrated that ERK activation is necessary for BDNF-related improvements in learning and long-term potentiation (Peng et al. 2010; Zhang et al. 2015a). There is a highly complicated relationship between SorLA and BDNF; as SorLA has the ability to promote the trafficking of TrkB receptors across the synaptic membranes and its deficiency can impair neuritic trafficking of TrkB and blunt response to BDNF (Rohe et al. 2013; Schmidt et al. 2017). Also, SorLA can be strongly induced when neurons are stimulated by BDNF (Rohe et al. 2013). It is noteworthy to mention that melatonin could enhance BDNF and pTrkB levels (Wei et al. 2015).

Previous work showed that LPS-induced deterioration in learning and memory is closely related to its ability to reduce the hippocampal pTrkB density and BDNF levels (Zhang et al. 2014; Wei et al. 2015). In the current study, the LPS-only group showed decreased levels of pTrkB, and BDNF expression and in line with previously documented work. Also, AGO had a direct impact on BDNF and pTrkB levels, and successfully reversed LPS negative impact on their levels but this action was partially diminished by PD98059. Another mechanism by which SorLA can protect neurons against LPS-induced amyloid beta deposition, loss of neuroplasticity, and neurotoxicity is by enhancing BDNF actions (Jiao et al. 2016).

Numerous neurological illnesses are strongly correlated with the dysregulation of presynaptic proteins like Synapsin I and synaptophysin, as well as postsynaptic proteins like PSD-95. In some pathological states, the level of those biomarkers is strongly correlated to the rate of amyloidogenesis (Tu et al. 2014; Liang et al. 2015; Maesako et al. 2019). Consistent with prior studies, LPS administration showed a dramatic reduction in hippocampal Synapsin I, synaptophysin, and PSD-95 expression levels, thus having a negative impact on synaptoplastic state, learning, and memory (Chen et al. 2017b; Khan et al. 2018; Zhu et al. 2019; Fang et al. 2020; Yang et al. 2020a; Sun et al. 2021). It was also noted that BDNF has the ability to improve plasticity; this action could be related to its ability to activate the ERK pathway, enhancing synapsin I, and Synaptophysin and PSD-95 expression (Tartaglia et al. 2001; Hu et al. 2011; Zhang et al. 2015b). Also, a previous study showed that soluble SorLA is able to enhance neurite regeneration via activating ERK pathway, and this effect was diminished in presence of PD98059 (Stupack et al. 2020). All of this was reflected in our results where AGO reversed LPS impact on Synapsin I, Synaptophysin, and PSD95 levels, and its positive impact was diminished by administering PD98059. It is noteworthy to mention that a previous study showed that melatonin could upregulate hippocampal presynaptic and postsynaptic proteins, thus improving neuroplasticity (Stefanova et al. 2015).

Autophagy is a strictly controlled intracellular that aids in the degradation and clearance of some cytoplasmic proteins, and certain pathogens through the autophagosome-lysosomal pathway (Cai et al. 2015). Normal physiological levels of autophagy are advantageous for neuronal survival because they can serve as a critical protective mechanism against stress stimuli, but excessive autophagy could be detrimental and result in autophagic cell death (Choi et al. 2013; Liu and Zhang 2017). Previous studies showed that autophagy is tightly connected to LPS-induced inflammation, by controlling and manipulating the inflammasome-dependent responses by triggering and enhancing the release of interleukin 1beta (IL-1β) and IL 18 (Liu and Zhang 2017; Iula et al. 2018; Claude-Taupin et al. 2018).

In line with a previous study, LPS showed elevated LC3b with increased Beclin1 and declined P62 levels, which indicate an active and sustained autophagic route (Ma et al. 2019). The relationship between Aβ and autophagy is extremely intricate. While some studies suggest that amyloid beta can be reduced and degraded by autophagy in a variety of systems, other findings suggest that Aβ can be generated in the autophagosomes and autophagic process could play a part in the Aβ extracellular secretion (Menzies et al. 2017). Furthermore, some studies revealed that Aβ deposition could be a direct enhancer of autophagy in a time-dependent manner, and neither autophagic inducers nor inhibitors could have an impact on Aβ clearance (Cai et al. 2015; Liu and Li 2019). This could be a possible explanation for the elevated Aβ levels in the group that received LPS only. In the current study, we found that AGO was able to halt this autophagic pathway, which was evident in the levels of autophagy biomarkers and was supported by previous work (Lan et al. 2022). Thus, we suggest that AGO could possibly have a time-dependent impact on regulating autophagy, resulting in a protection against the autophagic cell death induced by LPS. This protective action could be attributed to the BDNF direct effect on mTOR pathway via PI3K-Akt pathway, or through a complicated interaction between autophagy and ERK pathway (Smith et al. 2014; Chen et al. 2017a; Nikoletopoulou et al. 2017; Xie et al. 2018). The group that received PD98059 did not show any significant impact on autophagy biomarkers, thus supporting the idea of BDNF action on autophagy and the direct action of AGO on BDNF levels, but further studies are needed to detect the exact reason behind this complex interaction.

## Conclusion

All in all, AGO showed a promising effect in modulating the devastating action of LPS on neuroinflammation, compromised autophagy, and impaired neuroplasticity. AGO’s therapeutic advantages—which were associated with the previously evaluated biomarkers—were eliminated by using PD98059, except for the autophagy biomarkers which were not affected by administering the blocker; thus, suggesting that the autophagic modulatory action of is independent on the ERK pathway.

### Supplementary Information

Below is the link to the electronic supplementary material.

Supplementary file Fig. S1. Simplified version of the protocol design, including the medications that each group received throughout the experimental period. The training phase of MWM started from day 4 to day 7 followed by a probe test on day 8 before the scarification process. On the 8th day, rats were euthanized under light anesthesia, and the brains were then harvested. (JPG 103 KB)

FSupplementary file Fig. S2. Showing the cued trial which was carried out after the probe test, in order to rule out the impact of visual, motor, and motivational variation on the rats performance. In this test, there was no statistically significant difference in escape latencies between any of the groups. All values are presented as the mean ± S.D. and statistical analysis was carried out using one-way analysis of variance test (ANOVA) followed by Tukey’s test for multiple comparisons. The P-value for significance was set at p< 0.05. (JPG 64 KB)

## Data Availability

Data will be provided upon reasonable request.
