# Supplementary figures and images for: Agomelatine improves memory and learning impairments in a rat model of LPS-induced neurotoxicity by modulating the ERK/SorLA/BDNF/TrkB pathway
Source: Naunyn Schmiedebergs Arch Pharmacol. 2023 Sep 15;397(3):1701–14. doi: 10.1007/s00210-023-02717-w (PMC10858839; doi:10.1007/s00210-023-02717-w)

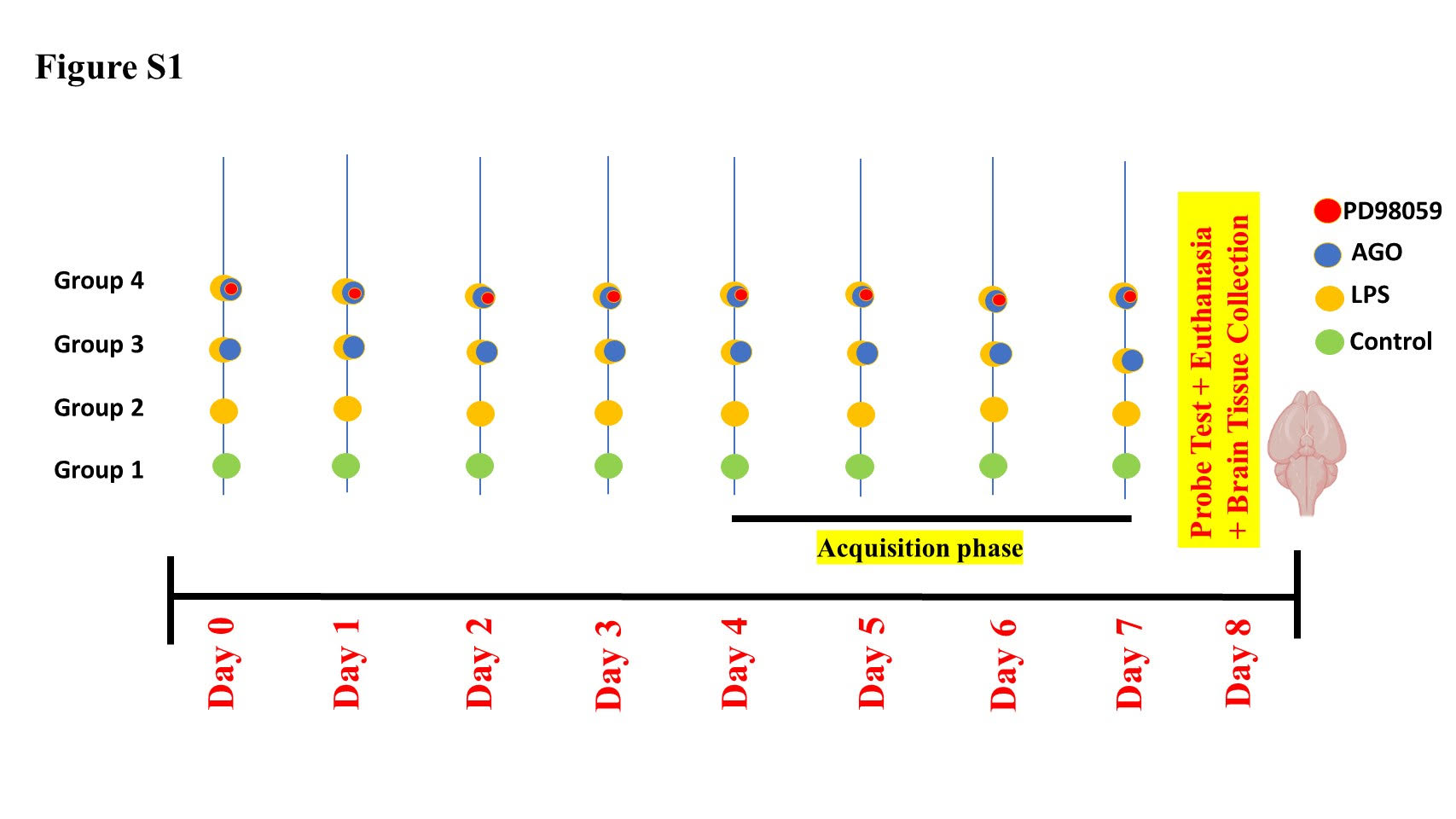

Supplement: Supplementary file 1 — Supplementary file Fig. S1. Simplified version of the protocol design, including the medications that each group received throughout the experimental period. The training phase of MWM started from day 4 to day 7 followed by a probe test on day 8 before the scarification process. On the 8th day, rats were euthanized under light anesthesia, and the brains were then harvested. (JPG 103 KB) [file 210_2023_2717_MOESM1_ESM.jpg]

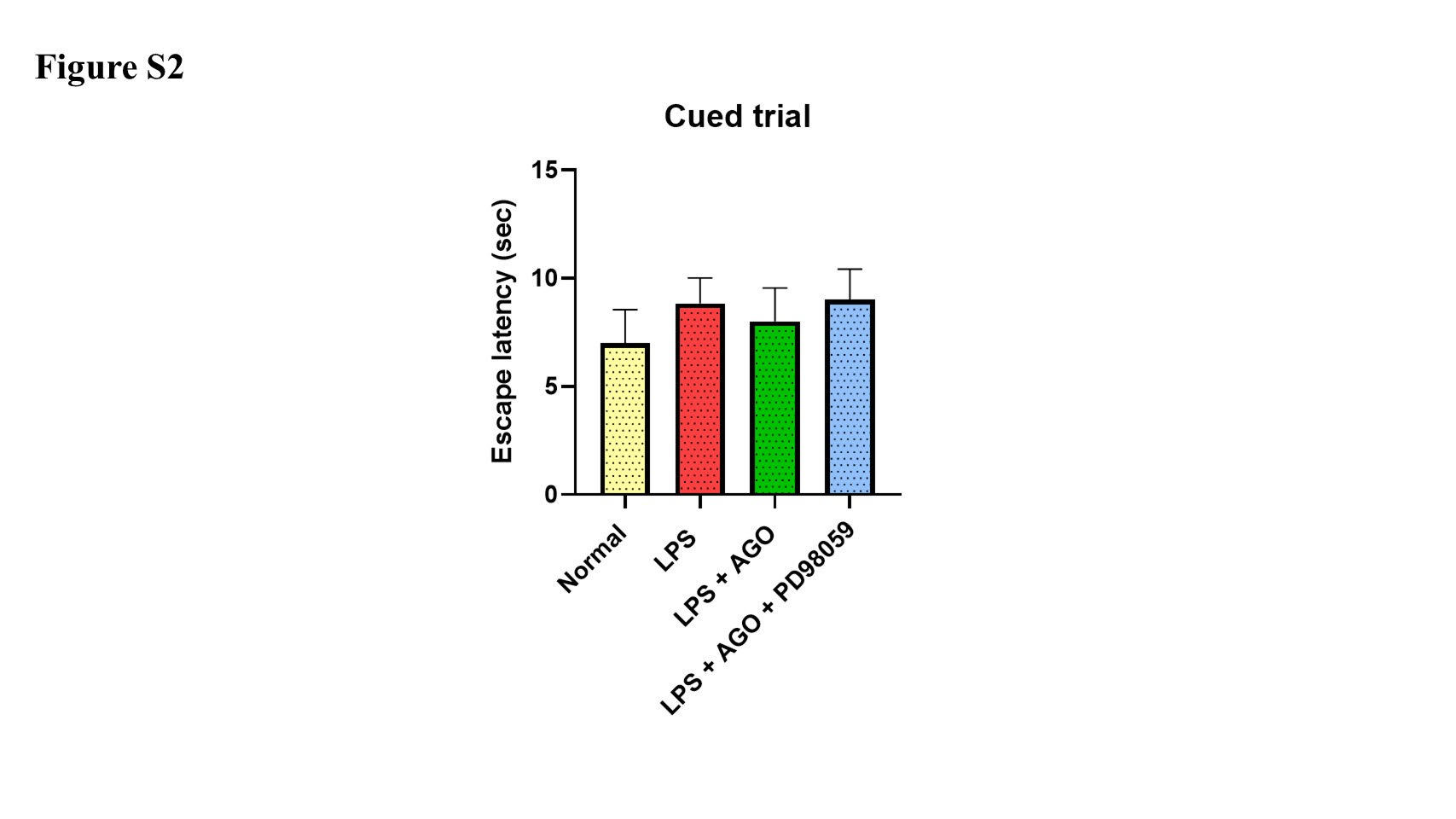

Supplement: Supplementary file 2 — FSupplementary file Fig. S2. Showing the cued trial which was carried out after the probe test, in order to rule out the impact of visual, motor, and motivational variation on the rats performance. In this test, there was no statistically significant difference in escape latencies between any of the groups. All values are presented as the mean ± S.D. and statistical analysis was carried out using one-way analysis of variance test (ANOVA) followed by Tukey’s test for multiple comparisons. The P-value for significance was set at p< 0.05. (JPG 64 KB) [file 210_2023_2717_MOESM2_ESM.jpg]
